# Salt Stress-Related Mechanisms in Leaves of the Wild Barley *Hordeum spontaneum* Generated from RNA-Seq Datasets

**DOI:** 10.3390/life13071454

**Published:** 2023-06-27

**Authors:** Aminah A. Barqawi, Aala A. Abulfaraj

**Affiliations:** 1 Department of Chemistry, Al-Leith University College, Umm Al-Qura University, Makkah 28434, Saudi Arabia; aabbarqawi@uqu.edu.sa; 2 Biological Sciences Department, College of Science & Arts, King Abdulaziz University, Rabigh 21911, Saudi Arabia

**Keywords:** photorespiration, ubiquitination, homeostasis, nitrate assimilation, carbon level

## Abstract

This study aims to detect salt stress-related genes and mechanisms of the wild barley *Hordeum spontaneum*. Among the generated RNA-Seq datasets, several regulated transcripts are influenced by levels of cellular carbon, nitrogen and oxygen. Some of the regulated genes act on photorespiration and ubiquitination processes, as well as promoting plant growth and development under salt stress. One of the genes, encoding alanine:glyoxylate aminotransferase (AGT), participates in signaling transduction and proline biosynthesis, while the gene encoding asparagine synthetase (ASN) influences nitrogen storage and transport in plants under stress. Meanwhile, the gene encoding glutamate dehydrogenase (GDH) promotes shoot and root biomass production as well as nitrate assimilation. The upregulated genes encoding alpha-aminoadipic semialdehyde synthase (AASAS) and small auxin-up RNA 40 (SAUR40) participate in the production of proline and signaling compounds, respectively, while the gene encoding E3 ubiquitin-protein ligase regulates the carbon/nitrogen-nutrient response and pathogen resistance, in addition to some physiological processes under biotic and abiotic stresses via signal transduction. The gene encoding the tetratricopeptide repeat (TPR)-domain suppressor of STIMPY (TSS) negatively regulates the carbon level in the cell. In conclusion, this study sheds light on possible molecular mechanisms underlying salt stress tolerance in wild barley that can be utilized further in genomics-based breeding programs of cultivated species.

## 1. Introduction

The impacts of global climate change and soil salinization, in addition to the worldwide demand for increasing crop yields due to the rapidly growing human population, mean it is mandatory for breeders to generate crops with increased levels of tolerance against harsh environmental conditions [1,2,3]. Wild relatives of cultivated plant species provide supplementary insights in breeding and genetic transformation programs towards the utilization of stress-specific genes to complement cultivated species, increasing their ability to tolerate salt stress and to promote plant growth [4]. The level of salt stress that is life-threatening to cultivated barley is estimated to be 100 mM NaCl [5]. *Hordeum spontaneum* is a wild barley species that is the ancestor of the cultivated diploid (2n = 2x = 14) barley species, *H. vulgare*. Cultivated barley is among the major cereal crops worldwide and is predominantly self-pollinated. However, it is possible to generate fertile progeny by crossing wild and cultivated barley [6]. This indicates the short genetic distance between the two barley species; a feature that is almost missing in bread (2n = 6x = 42), durum (2n = 4x = 28) and wild (2n = 2x = 14) wheats. However, genetic differences as well as differential controlling elements between the two barley species exist. These differences include stress-related genes that can be targets for subsequent breeding programs, not only in barley but also in many other field crops via metabolic engineering approaches. Previous reports indicate that this wild barley includes a large number of disease resistance and abiotic stress tolerance genes [7,8,9,10,11,12]. More recently, Liu and colleagues [13] further indicated the existence of ~1300 gene families in this wild barley that are missing in cultivated barley as well as other cereal crops.

In the genomic era, high-throughput sequencing methods provide new insights into the signatures of wild plant genomes, offering potential candidates for field crop improvement [14,15]. Therefore, understanding the molecular dynamics of salt stress responses will allow the development of genotypes with enhanced performance and the ability to manage programmed cell death mechanisms under stress conditions [16,17,18,19]. Next-generation sequencing (NGS) represents the latest tool for studying transcriptomes as well as the physiological and biochemical mechanisms of plant salt stress tolerance, whether or not genome sequencing data is available, such as *H. spontaneum* [13,20,21]. These tolerance mechanisms include the regulation of ion homeostasis, osmotic potential, plant hormone signaling responses, ubiquitination and cell wall composition [3,22,23]. A major influencer of salt stress is the sodium ion, whose high concentration hinders water availability and the plant’s ability to absorb soil nutrients [24]. Thus, an ion transport approach might be a candidate for manipulating the plant’s ability to withstand salt stress.

The present work aims to study the transcriptome of wild barley (*H. spontaneum*) leaves under salt stress exposure over time in order to obtain an overview of the specific molecular mechanisms used by this wild plant species to tolerate salt stress.

## 2. Materials and Methods

### 2.1. Salt Stress Experiment and Isolation of RNA

Plantlets of wild barley (*H. spontaneum*) were grown as previously described [25]. Fourteen-day-old seedlings were salt-treated at 500 mM NaCl for 0 (used as a control), 2, 12 and 24 h in a replicated experiment. Total RNAs were isolated from the fine-powdered leaves as previously described [25]. Then, the purified RNA samples (30 µg at 400 ng/µL) were sent to Beijing Genomics Institute (BGI), China, for deep sequencing using an Illumina MiSeq platform.

### 2.2. RNA-Seq Analysis

The raw data recovered from next generation sequencing were submitted in FASTQ format to NCBI and the experiment received an accession number of PRJNA227211. Then, the raw data were filtered and trimmed as described [26]. Then, the clean raw data was de novo assembled via Trinity as described [25]. Assembly of the transcript contigs resulted in the identification of transcripts using an ORF-Predictor [27], while EdgeR (version 3.0.0) was used to detect differentially expressed (DE) transcripts and to make a cluster analysis. A Blastx was performed and the fold change of the DE transcripts was estimated using default parameters [25,28]. Then, a significant Pearson correlation was measured in order to generate a heat map and GO terms were determined using Blast2GO. The coding sequences (CDS) were categorized with a WEGO analysis, and the Protein Information Resource (PIR) was utilized to determine the UniProt IDs. Then, the predicted CDS were subjected to annotation in order to detect the function of the DE transcripts. The data of the RNA-Seq was validated using real-time PCR for four regulated transcripts (Table S1) and their expression levels were calculated relative to the barley *actin* house-keeping gene as previously described [28].

## 3. Results

### 3.1. Quality Assurance and RNA-Seq Data Validation

The raw data recovered from the leaves of *H. spontaneum* transcriptomes were previously analyzed against the barley UniGene database available at the NCBI [28]. However, we followed a genome-guided assembly approach to secure coverage of all genes under the adverse conditions. The resulting number of DE transcripts in the previous study with a fold change (FC) of ≤ 2 was 9277, of which only 3861 transcripts were mapped against the *H. vulgare* reference genome. However, the genome-guided assembly approach with a similar FC level resulted in the occurrence of ~11,000 differentially expressed transcripts (Table S2).

**Figure 1:**
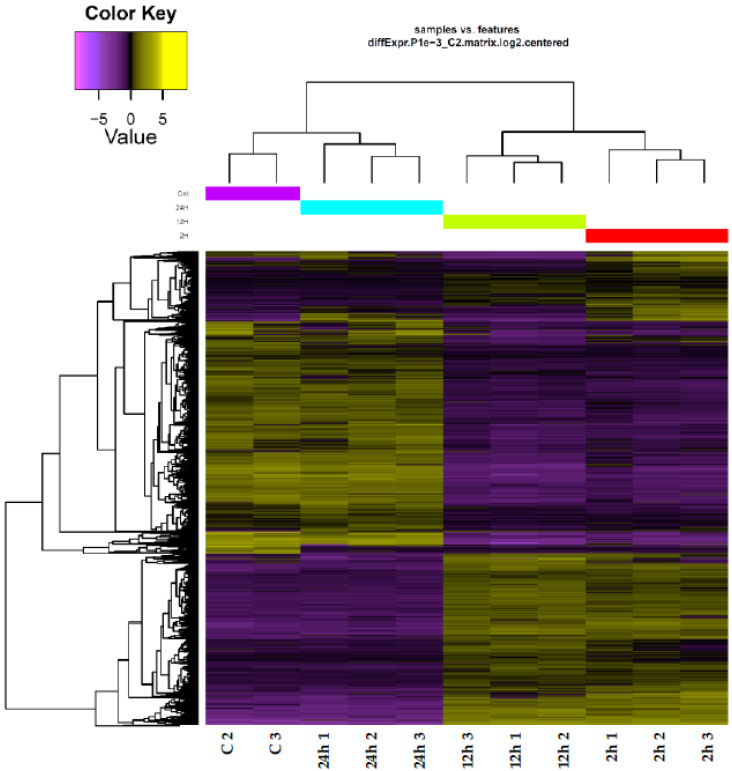
Heat map describing the interrelation among genes at control (C) and salt-stressed (500 mM NaCl) samples across different timepoints (e.g., 2, 12 and 24 h) in leaves of *H. spontaneum*.

The heat map to detect transcriptome patterns under salt stress showed that the replicates at each timepoint of salt stress are closely related (Figure 1). The heat map also indicated a close relationship between the transcriptomes of leaves exposed to 0 and 24 h of salt stress on one hand, and between transcriptomes of leaves exposed to 2 and 12 h on the other. These results reflect the instant response of the plant to salt stress that mostly diminishes after prolonged exposure, e.g., 24 h. We chose a salt concentration of 500 mM NaCl as this wild plant grows naturally on the Red Sea where the salt concentration of the water is much higher; thus, we expected the plants to be able to tolerate this level.

**Figure 2:**
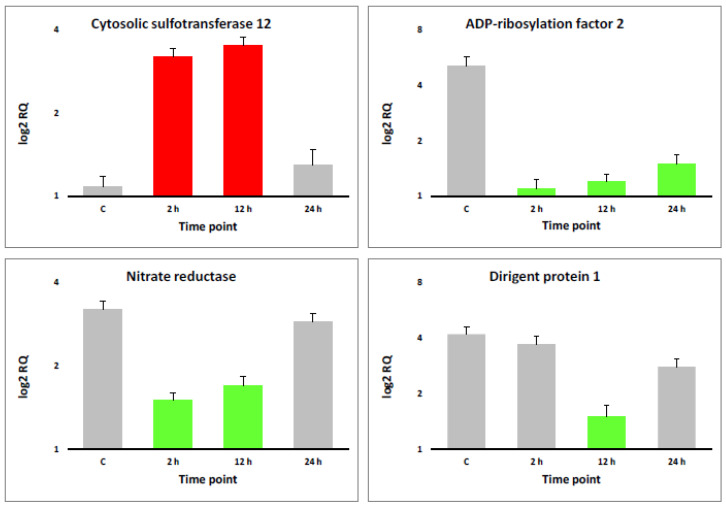
qPCR used for validating RNA-Seq datasets in leaves of *H. spontaneum* under salt stress (500 mM NaCl) using four randomly selected transcripts that were upregulated at the 2 and 12 h timepoints (cytosolic sulfotransferase 12). Meanwhile, downregulation occurred at the 2, 12 and 24 h timepoints (ADP-ribosylation factor 2); at the 2 and 12 h (nitrate reductase); and at the 12 h timepoint (dirigent protein 1). Upregulated transcripts are shown in red columns, while downregulated transcripts are shown in green columns. Unregulated transcripts are shown in gray columns.

Validation of RNA-Seq datasets was performed via real-time PCR for four transcripts, e.g., one was upregulated at 2 and 12 h of salt stress (cytosolic sulfotransferase 12 of cluster 17), the second was downregulated at 2, 12 and 24 h (ADP-ribosylation factor 2 of cluster 19), the third was downregulated at 2 and 12 h (nitrate reductase of cluster 25) and the fourth was downregulated at 12 h (dirigent protein 1 of cluster 30). Figure 2 indicates that the results of the real-time PCR fully support those of the RNA-Seq datasets introduced in Tables S2–S4.

### 3.2. GO Classification

**Figure 3:**
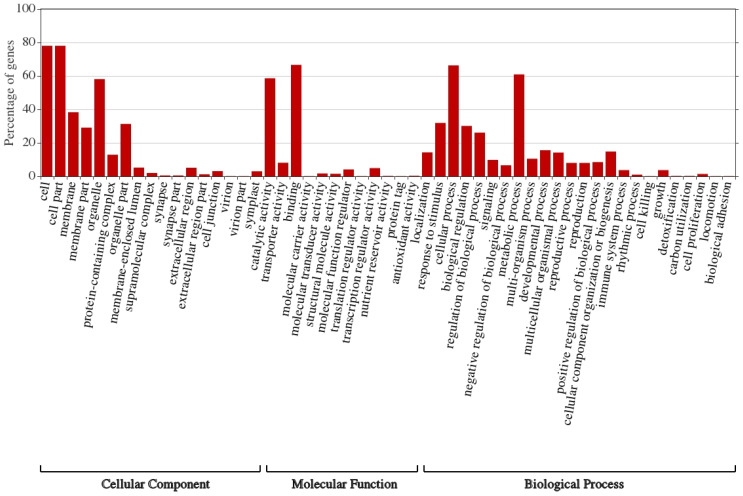
GO classification of upregulated genes under salt stress (500 mM NaCl) based on the similarity search within leaf CDS in the leaf transcriptome of *H. spontaneum*.

**Figure 4:**
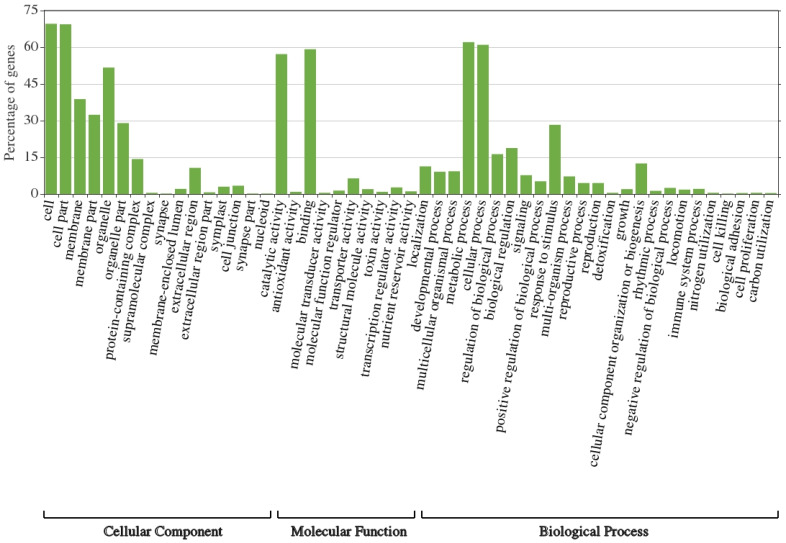
GO classification of downregulated genes under salt stress (500 mM NaCl) based on the similarity search within leaf CDS in the leaf transcriptome of *H. spontaneum*.

The annotated transcripts in the leaves of wild barley under salt stress were assigned to three main categories: “cellular component”, “molecular function” and “biological process” (Figure 3 and Figure 4). Highly enriched subcategories of “cellular component” included “cell”, “cell part”, “membrane”, “membrane part”, “organelle” and “organelle parts”; for “molecular function” they included “catalytic activity” and “binding”; and for “biological process” they included “response to stimulus”, “cellular process”, “biological regulation”, “regulation of biological process” and “metabolic process” (Figure 3). Interestingly, highly suppressed subcategories due to salt stress in the three categories included the same subgroups (Figure 4). This indicates that large number of gene families in these subcategories of the three categories showed differential expression under salt stress.

### 3.3. Cluster Analysis

**Figure 5:**
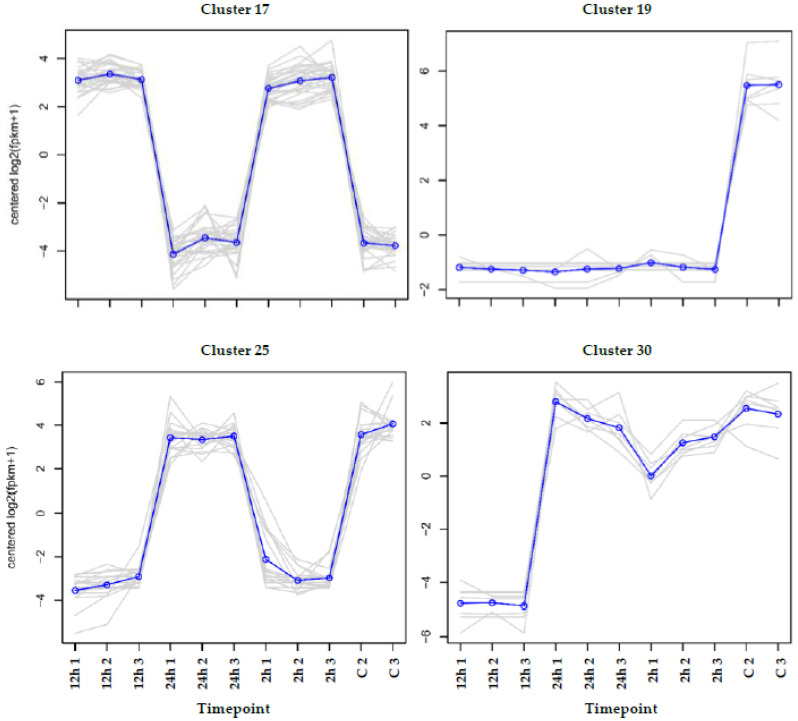
Examples of clusters that were upregulated under salt stress (500 mM NaCl) at the 2 and 12 h timepoints (cluster 17), while being downregulated at the 2, 12 and 24 h timepoints (cluster 19), at the 2 and 12 h timepoints (cluster 25) and at the 12 h timepoint (cluster 30) in the leaf transcriptome of *H. spontaneum*. Detailed descriptions of regulated transcripts of these clusters are shown in Tables S2 and S3.

**Figure 6:**
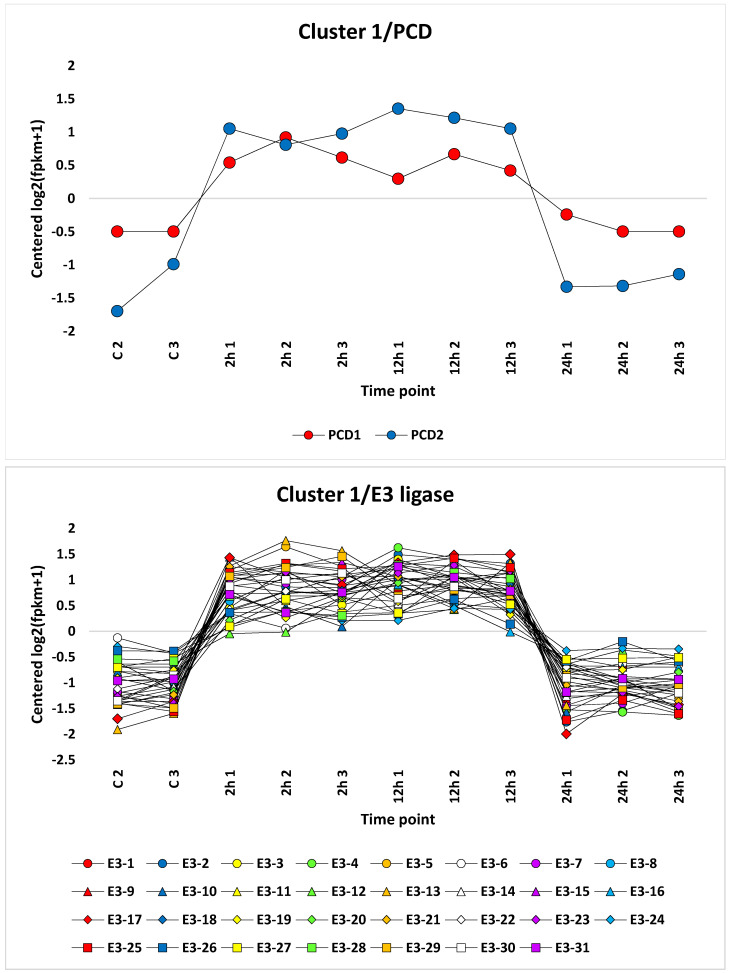
Expression patterns of coexpressed genes encoding E3 ubiquitin-protein ligase and those related to programmed cell death of cluster 1 for samples treated with salt stress (500 mM NaCl) at the 0, 2, 12 and 24 h timepoints in the leaf transcriptome of *H. spontaneum*. Descriptions of the genes are shown in Tables S2 and S4.

The analysis of differentially expressed transcripts, either annotated or unannotated, due to salt stress resulted in the recovery of 69 clusters with appropriate algorism (Table S2, Figure S1). Among these clusters, some transcripts in eight clusters were selected for further analysis (Table S3). The criteria for selecting clusters were based on the feasibility of annotation data, fold change and concordance of expression levels for transcripts in a single cluster across different timepoints. The selected expression patterns included upregulation under salt stress (500 mM NaCl) at the 2 and 12 h timepoints (clusters 17, 23 and 39); downregulation at the 2, 12 and 24 h timepoints (cluster 19); downregulation at the 2 and 12 h timepoints (clusters 25, 36 and 7); and downregulation at the 12 h timepoint (cluster 30). These clusters included 280 differentially expressed genes, of which 177 were annotated and hold differential expression levels of ≥2 fold change. Examples of clusters showing these expression patterns are shown in Figure 5. In addition, selected coexpressed genes of cluster 1 with upregulation at the 2 and 12 h timepoints of salt stress were also analyzed (Figure 6). They included 31 genes encoding E3 ubiquitin-protein ligase enzymes and two coexpressed genes promoting programmed cell death (Table S4). 

## 4. Discussion

Upregulation at the 2 and 12 h timepoints of salt stress indicates the response of genes whose expression is required early after the occurrence of salt stress and only for few hours, after which the cell starts to adapt and no longer maintains this high rate of expression. This provides a chance for other genes, whose expression is required at later stages, to be expressed. Downregulation at the 2 and 12 h timepoints or at the 2, 12 and 24 h timepoints of salt stress indicates that these genes are required to be expressed at lower rates at two or all of the timepoints, as they might participate in a specific route within a pathway that is not required under stress conditions during these time windows. This might result in the blocking of such a route in the pathway. If the route is bidirectional, then the downregulated transcripts might occasionally force the pathway to favor the opposite direction. Regulation of these genes most likely occur in metabolic pathways whose metabolites are required at certain levels to maintain tissue, protein or hormone homeostasis and, consequently, growth rate.

**Figure 7:**
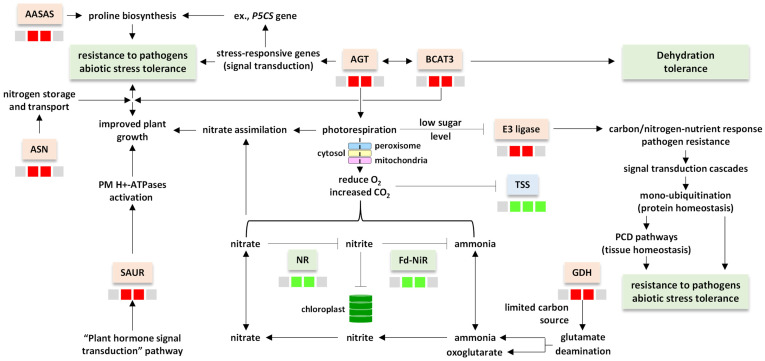
Influence of regulated genes in leaves of *H. spontaneum* under salt stress (500 mM NaCl) on several biological processes in terms of plant growth and development. The four-box pattern refers to regulated genes at the 0, 2, 12 and/or 24 h timepoints of salt (500 mM NaCl). Upregulated transcripts are shown in red boxes, downregulated transcripts are shown in green boxes and unregulated transcripts are shown in gray boxes. ASN = asparagine synthetase, AASAS = alpha-aminoadipic semialdehyde synthase, P5CS = delta1-pyrroline-5-carboxylate synthase, AGT = alanine:glyoxylate aminotransferase, BCAT3 = branched-chain-amino-acid aminotransferase 3, E3 ligase = E3 ubiquitin-protein ligase, PM = plasma membrane, TSS = tetratricopeptide repeat (TPR)-domain suppressor of STIMPY, SAUR = small auxin-up RNA 40, NR = nitrate reductase, Fd-NiR = ferredoxin-nitrite reductase, GDH = glutamate dehydrogenase, PCD = programmed cell death.

The selected genes for discussion include those encoding aminotransferases, of which two genes were upregulated after 2 and 12 h of salt stress (cluster 23, Table S3). Aminotransferases were cited earlier to participate in the regulation of biotic and abiotic stresses [29]. The first enzyme, namely, alanine:glyoxylate aminotransferase (AGT) (Table S3), participates in photorespiration in resistance to several plant pathogens, as well as in salt and water deficit tolerance [29,30]. Meanwhile, the second enzyme, namely, branched-chain-amino-acid aminotransferase 3 (BCAT3) participates in the accumulation of BCAA and the enhanced tolerance against dehydration stress (Figure 7) [31,32,33]. Overexpression of the gene encoding AGT in rice induced several other genes crucial in signaling transduction pathways [29]. These genes include *RD22, COR47, ADH1, RAB18* and *P5CS1* [34]. The latest gene, *P5CS1*, encodes a bifunctional enzyme, namely, delta1-pyrroline-5-carboxylate synthase 1 (P5CS1) with a major role in proline synthesis to confer stress tolerance (Figure 7) [35]. Branched-chain-amino-acid aminotransferase 3 (BCAT3) exists in chloroplasts and is effective in restoring the growth rate under stress conditions (Figure 7) [36].

Photorespiration involves a battery of enzymes for exchanging metabolites between chloroplasts, leaf peroxisomes and mitochondria [34]. We speculate that this is a transport process to neutralize the influence of stress at the level of cell organelles (Figure 7). In C_3_ plants, photorespiration reduces the efficiency of photosynthesis by 25% [37]. However, engineered plants with reduced photorespiration rates did not result in higher plant growth rates. Thus, photorespiration is not per se a harmful process to the organism. In addition, it was proven that photorespiration participates in nitrate assimilation as the main nitrogen source in cells [38]. Ammonia-to-nitrate is a reversible two-step reaction, as ammonia can be oxidized to nitrite, which in turn is oxidized to produce nitrate that becomes amenable to assimilation by plant roots (Figure 7). Upon a shortage of O_2_, this reaction can be reversed in which nitrate is reduced to nitrite via the action of the gene encoding nitrate reductase (NR), then ammonia is produced via the action of the gene encoding ferredoxin-nitrite reductase (Fd-NiR). We speculate that the direction of the reaction is influenced by the level of O_2_ in the cell. Interestingly, genes encoding NR and Fd-NiR in the present study showed exactly opposite rates of expression to that encoding AGT, as their expression was reduced after 2 and 12 h of salt stress (Table S3). This indicates that Nr and Fd-NiR genes participate at one end of a cascade of reversible events starting with the influence of salt stress to enrich AGT, which in turn promotes photorespiration that induces the reduction of the O_2_ level in cells. A low level of O_2_ might be the reason for the downregulation of the two genes encoding NR and Fd-NiR, which makes the nitrate-to-ammonia direction unfavorable (Figure 7). Furthermore, earlier reports indicate that the elevated CO_2_ that accompanies a shortage of O_2_ due to photorespiration suppresses nitrite transport into chloroplasts [39].

Accordingly, we assume that the increase in photorespiration rate under salt stress due to the action of the gene encoding AGT in *H. spontaneum* will shorten supply of O_2_. Those cells in favor of converting ammonia to nitrate are then redirected towards nitrate assimilation via manipulation of a number of genes in a cascade of events (Figure 7). The asparagine synthetase gene, *ASN*, was thought to respond to salt and osmotic stresses [40]. This gene is upregulated at the 2 and 12 h timepoints of salt stress (Table S3). Free asparagine has a vital role in both the storage and transport of nitrogen [41]. Both asparagine and proline were reported to accumulate in *Hordeum* and wheat as a response to salt stress and participate in conferring stress tolerance [42,43]. Accumulated evidence in the present study, in terms of AGT and ASN upregulation as well as NR and Fd-NiR downregulation, align with the cited influence of these genes on several biological processes in the cell, including the response to adverse environmental conditions (Figure 7) [34,44]. Asparagine is also reported to play a major role in nitrogen storage and transport, as it contains the highest nitrogen to carbon ratio among different amino acids [45,46,47]. Interestingly, the transport of asparagine promotes sequential nitrogen mobilization from the source, namely the xylem/phloem, to be stored in the ultimate sink, namely the seed, to secure seed-filling under stress conditions [43,48].

Glutamate dehydrogenase (GDH) is among those enzymes whose encoding gene is upregulated in leaves of *H. spontaneum* at the 2 and 12 h timepoints of salt stress (Table S3). Under salt stress, transgenic tobacco overexpressing the gene encoding this enzyme resulted in higher shoot and root biomass production [49]. This is accompanied by differential accumulation of several carbon and nitrogen containing molecules, such as digalactosylglycerol, erythronate and porphyrin. This suggests the possible contribution of these molecules to improving the performance of plants under salt stress. GDH has a central position in carbon and nitrogen metabolism, but it favors deaminating glutamate and the production of oxoglutarate and ammonia when the carbon source is limited [50]. Under such conditions, ammonia can proceed in the oxidation reaction towards nitrate assimilation, which is the major route for incorporating ammonia via the action of AGT as previously noted (Figure 7) into useful organic molecules such as nitrates. This conclusion in the leaves of *H. spontaneum* aligns with those previously described [51].

Genes encoding E3 ubiquitin-protein ligases were upregulated in the leaves of *H. spontaneum* at the 2 and 12 h timepoints of salt stress (Tables S3 and S4). The encoded enzymes were reported to regulate the carbon/nitrogen-nutrient response and pathogen resistance in plants, as well as help adjust physiological processes under biotic and abiotic stresses via signal transduction cascades. Signal transduction events facilitate various cellular responses including ubiquitination [22]. Ubiquitination, e.g., a post-translational modification, acts in mediating growth under both normal and adverse environmental conditions [52]. Ubiquitin is a protein that is stable, highly conserved and universally expressed. E3 ligase facilitates the transfer of ubiquitin to the target protein (mono-ubiquitination). Guo et al. [53] indicated that overexpression of a single mono-ubiquitin gene such as E3 ligase can enhance tolerance to multiple stresses. Martin et al. [54] also indicated that plants grown under high sugar and low nitrogen levels experience arrested post-germination growth and cannot survive under such stress conditions due to the influence of one type of E3 ubiquitin-protein ligase, namely RING-type. Photorespiration is known to reduce the efficiency of photosynthesis and, consequently, reduces the sugar level in plant leaves. Therefore, AGT can alleviate the influence of E3 ligase, as AGT promotes photorespiration which serves in avoiding high sugar levels. This enables plants to progress through post-germination growth stages and survive under stress conditions. Extensive exposure to salt stress mediates two types of homeostasis: tissue homeostasis via programmed cell death (PCD) and protein homeostasis via ubiquitination. Under salt stress, PCD acts on eliminating damaged and unwanted cells [55], while ubiquitination acts on eliminating damaged and unwanted proteins in the cell [56]. Such post-translational modification of proteins signals the battery of PCD pathways if the intensity of protein damage in the cell is high (Figure 7) [55]. 

Genes encoding alpha-aminoadipic semialdehyde synthase (AASA synthase or AASAS) and small auxin-up RNA 40 (SAUR40) were also upregulated at the 2 and 12 h timepoints of salt stress (Table S3). The AASA synthase enzyme is bifunctional in the “lysine degradation” or “saccharopine” pathway as it catalyzes the first two steps in the pathway. The enzyme converts the amino acid lysine to α-aminoadipic-δ-semialdehyde (AASA) which is subsequently converted to aminoadipic acid (AAA) [57]. The overexpression of the gene encoding AASAS is proven to participate in the abiotic stress response and tolerance. The authors justified tolerance by the production of osmolytes, e.g., proline (Figure 7), or signaling compounds that induce downstream stress-responsive genes. The SAUR family is implicated in a wide range of developmental and physiological processes, such as activation of H+-ATPases in plasma membranes and the promotion of cell growth via the “plant hormone signal transduction” pathway [58]. The authors indicated that the expression of genes encoding SAURs is also regulated by other hormones and signals that promote plant growth and development.

The tetratricopeptide repeat (TPR)-domain suppressor of STIMPY (TSS) and ADP-ribosylation factor 2 (ARF2) are two proteins encoded by genes that are downregulated under stress conditions in the leaves of *H. spontaneum* at the 2, 12 and 24 h timepoints of salt stress (Table S3). The first protein, e.g., TSS, negatively regulates the proliferation of meristematic tissue by integrating developmental signals with carbon source availability [59]. Under stress conditions, the carbon source ought to increase due to photorespiration, as previously noted. According to Skylar et al. [59], the expression of the gene encoding the TSS protein negatively correlates with the carbon level; thus, it is likely that this gene will be downregulated in alignment with the results of the present study. The ARF2 protein was reported to accumulate under salinity stress [60]. In disagreement with our results, Joshi et al. [61] indicated that the overexpressing of the *ARF1* gene in rice and Arabidopsis results in higher salt and drought tolerance, whereas the *ARF2* gene in the present study was downregulated (Table S3). However, genomic analysis on the structure, organization and evolution of *ARF* genes by Joshi et al. [61] questioned the aforementioned expression profile in response to both stresses. Therefore, we call for further experimentation and debate in order to delineate the precise role of this gene family under stress conditions.

**Figure 8:**
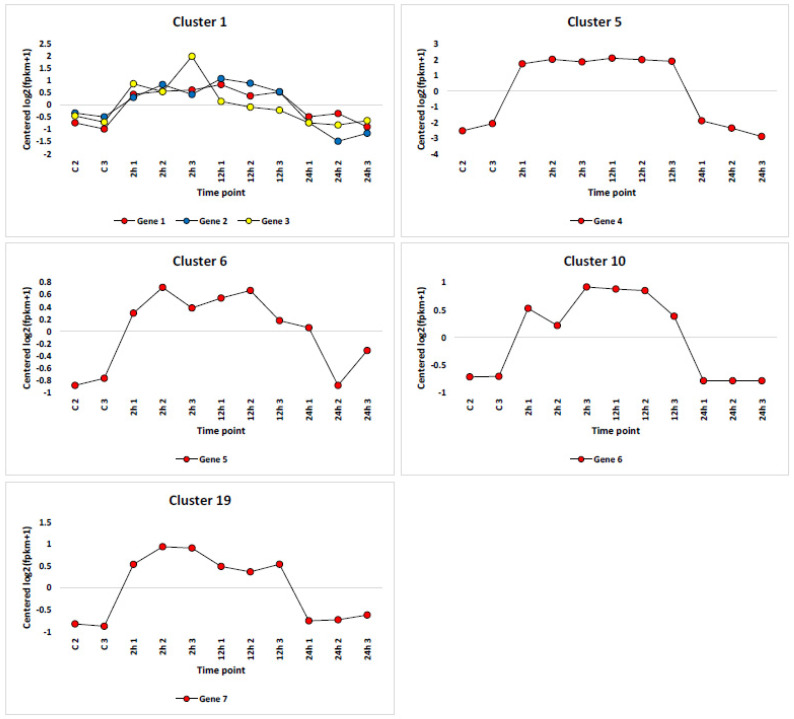
Expression patterns of genes specific to wild barley *H. spontaneum* in pathways “plant hormone signal transduction” (ko04075) and “MAPK signaling pathway-plant” (ko04016) at the 0, 2, 12 and 24 h timepoints of salt stress (500 mM NaCl). All genes showed upregulation at the 2 and 12 h timepoints of salt stress (500 mM NaCl). Gene 1 = encodes two-component response regulator ORR3, gene 2 = encodes serine/threonine-protein kinase BSK1-2, gene 3 = encodes auxin-responsive protein SAUR71, gene 4 = encodes auxin-responsive protein SAUR36 (isoform 1), gene 5 = encodes auxin-responsive protein SAUR36 (isoform 2), gene 6 = encodes auxin-responsive protein SAUR40, gene 7 = encodes protein phosphatase 2C. Genes 1-6 regulate enzymes/proteins of the “plant hormone signal transduction” pathway, while genes 2 and 7 regulate enzymes of “MAPK signaling pathway-plant”. These seven genes were recently proven to be regulated in wild barley (*H. spontaneum*), yet are not regulated in cultivated barley [13]. See Table S2 for more details.

Prior reports comparing wild (*H. spontaneum*) and cultivated (*H. vulgare*) barley indicate differences in phenotypic characteristics due to the different genetic makeup of the two genotypes [6,13]. Other reports indicate that less than half of the alleles found in wild barley exist in cultivated barley [62,63]. This differential presence/expression of alleles include those related to biotic [7,8] and abiotic [11,12] stresses. More recently, Liu and colleagues [13] detected some abiotic stress-related genes that are enriched only in wild barley. In the present study, we can confirm enrichment of seven of these abiotic stress-related genes, which encode enzymes/proteins of two pathways, namely, “plant hormone signal transduction” and “MAKP signaling pathway-plant” at the 2 and 12 h timepoints of salt stress (Figure 8). Of these, genes and gene isoforms encoding SAUR36, SAUR40 and SAUR72 directly promote plant growth and development of wild barley under stress [58]. The lack of enrichment in this SAUR protein family in cultivated barley might explain its inability to withstand salt stress, as SAUR proteins represent the end product of this signal transduction avenue. The other wild barley-specific gene encoding the two-component response regulator, ORR3, was recently reported to confer dehydration tolerance in cyanobacteria [64]. Meanwhile, the gene encoding serine/threonine-protein kinase BSK1-2 regulates innate immunity in plants [65], and the gene encoding the protein phosphatase 2C has influences on metabolism, hormone levels and plant growth factors under salt stress, according to Chu [66,67]. Collectively, these seven genes add to our understanding of the differential performance of the two genotypes under salt stress.

## 5. Conclusions

The present study has expanded our knowledge of molecular processes of salt tolerance in wild barley (*Hordeum spontaneum*). Under stress conditions, we paid attention to the genes regulating the levels of sugar and CO_2_ on one hand, and those regulating O_2_ and nitrogen (particularly nitrate) on the other. The levels of these elements and the molecules they generate contribute to the plant’s ability to grow and withstand abiotic stresses. This information can provide valuable insights when producing crop plants with improved salt tolerance.

## Data Availability

The raw next generation sequencing data were submitted in FASTQ format to NCBI and the experiment received accession number of PRJNA227211.

## Supplementary Materials

The following supporting information can be downloaded at: https://drive.google.com/drive/folders/1ZqUBMNx-o1HIf9fFhHF_IK9PB-9D-weI?usp=sharing.
